# Chromosome-level genome assembly of *Dynastes reidi* reveals structural evolution of autosomes and the sex chromosomes in Hercules beetles

**DOI:** 10.1093/g3journal/jkaf198

**Published:** 2025-08-22

**Authors:** My-Hanh Le, Max Proctor, Jen-Pan Huang

**Affiliations:** Biodiversity Research Center, Academia Sinica, Taipei 11529, Taiwan; Division of Biological Sciences, University of Montana, Missoula, MT 59812, United States; Biodiversity Research Center, Academia Sinica, Taipei 11529, Taiwan

**Keywords:** genome assembly, Giant Hercules beetle, *Dynastes reidi*, sex-linked region, sex chromosome identification

## Abstract

The Hercules beetles have long been iconic symbols of evolutionary diversification, sexual selection, and systematics. Despite their rapid phenotypic evolution and a rich history of inspiring evolutionary biologists, genomic resources for these charismatic beetles remain limited, especially for the Giant Hercules beetles. We present the first chromosome-level genome assembly of a Giant Hercules beetle from the Lesser Antilles. The assembled genome is approximately 837 Mb in size, with a scaffold N50 of 66.68 Mb, which can be anchored to 11 pseudochromosomes with a BUSCO completeness score of 95.9%. An estimate of 55.5% of the genome can be attributed to repetitive elements. Additionally, we detected candidate sex-linked chromosomes by comparing sequencing read depths between 1 male and 2 females using Illumina short reads. The chromosome-level genome assembly of *Dynastes reidi* not only provides critical insights into evolutionary and functional genomics but also supports informed conservation and management efforts. In addition, this genomic resource will enable future pangenome analyses aimed at understanding the genetic basis of species divergence and morphological innovation in beetles. Our study also marks the emergence of a new model system to investigate the origin and diversification of phenotypic novelty by leveraging genomic resources across diverse domesticated beetle breeds.

## Introduction

The Hercules beetles are large and charismatic scarab beetles that exhibit exaggerated male secondary morphologies, i.e. horns, which vary not only between species but also within species (Endrödi 1947; Dechambre 1980; Chalumeau and Reid 2002; Huang 2016, 2017; Huang and Knowles 2016). Additionally, the elytral color of the adult beetles can change in response to different humidity levels, putatively serving a camouflage function (Hinton and Jarman 1973). Variation in elytral coloration is evident both among individuals of the same species and between species (Huang 2016). Because of their charismatic features, Hercules beetles have become iconic models for systematic and evolutionary biology studies (Huang and Knowles 2016; Le et al. 2024). Recent research has unraveled that the structure of sex chromosomes in beetles, particularly in scarab beetles that include Hercules beetles (Dutrillaux and Dutrillaux 2013), can evolve rapidly. This discovery offers a unique perspective on the dynamic nature of sex chromosome evolution and opens up new opportunities to study the mechanisms underlying rapid changes in sex-determination systems in nature (Bijl and Mank 2025). Furthermore, advancements in the pet insect industry over the past 30 yr have taken advantage of this great phenotypic diversity to create distinct breeds of Hercules beetles that show unique body coloration or extremely long or thick male horn phenotypes (Lai and Ko 2001). These readily available phenotypic variants in domestic animals can be an unprecedented resource for understanding how novel phenotypes evolve and identifying the genomic regions that are responsible for their origins (such as the Piebaldism in domestic python breeds; Garcia-Elfring 2023).

*Dynastes reidi* Chalumeau, 1977 is an endemic Hercules beetle species from the island of Saint Lucia in the Lesser Antilles (Huang 2017). The Hercules beetles are composed of 2 species groups: the Giant Hercules group, which is characterized by its large body size and exaggeratedly long male horn, and the White Hercules group, which refers to their generally lighter or whitish body coloration. The species *D. reidi* is unique among others in the Giant Hercules group due to the lack of a major male phenotype. For example, most Giant Hercules beetles, including its sister species, *Dynastes hercules*, from the islands of Guadeloupe and Dominica, exhibit dimorphic major and minor male horn phenotypes; however, such dimorphism is less apparent in the White Hercules beetles (Chalumeau and Reid 2002; Huang 2016, 2017). Note that the loss of the major male horn phenotype is not uncommon among scarab beetles, which can occur rapidly even between populations of the same species (Huang and Morgan 2021). The size and shape of the male secondary sexual structures have also been heavily selected among pet insect breeds (Lai and Ko 2001), indicating their potential for rapid evolution. Nevertheless, *D. reidi* represents a fine empirical example to examine the genomic basis of repeated or paralleled losses of a phenotype that may have specific ecological significance—i.e. male–male competition and antipredator behavior (Beebe 1947; Jarman and Hinton 1974).

In this study, we generated a chromosome-scale genome assembly of *D. reidi*. To achieve this, we extracted genomic DNA from a single male specimen and applied a combination of complementary sequencing technologies: Oxford Nanopore sequencing to obtain ultralong reads, Illumina NovaSeq for high-accuracy short reads, and Hi-C sequencing to capture chromatin interaction patterns for scaffolding and genome architecture reconstruction. In addition, gene predictions and protein annotations for the newly assembled *D. reidi* genome were performed with RNA-seq data from the same individual. We further compared genome resequencing coverage between 1 male and 2 female *D. reidi* individuals to identify sex chromosomes, or sex-linked genomic regions, in the *D. reidi* genome. Specifically, our results contribute to the understanding of sex chromosome evolution (van der Bijl and Mank 2025), particularly in light of evidence suggesting that some species within this clade harbor neo-XY sex chromosome systems (Dutrillaux and Dutrillaux 2013). These systems are of special evolutionary interest, as they represent dynamic stages of sex chromosome differentiation and turnover, which offer a unique opportunity to investigate the genomic causes and consequences of sex chromosome evolution.

## Materials and methods

### Sample collection, genomic extraction, and genome sequencing

**Fig. 1.**
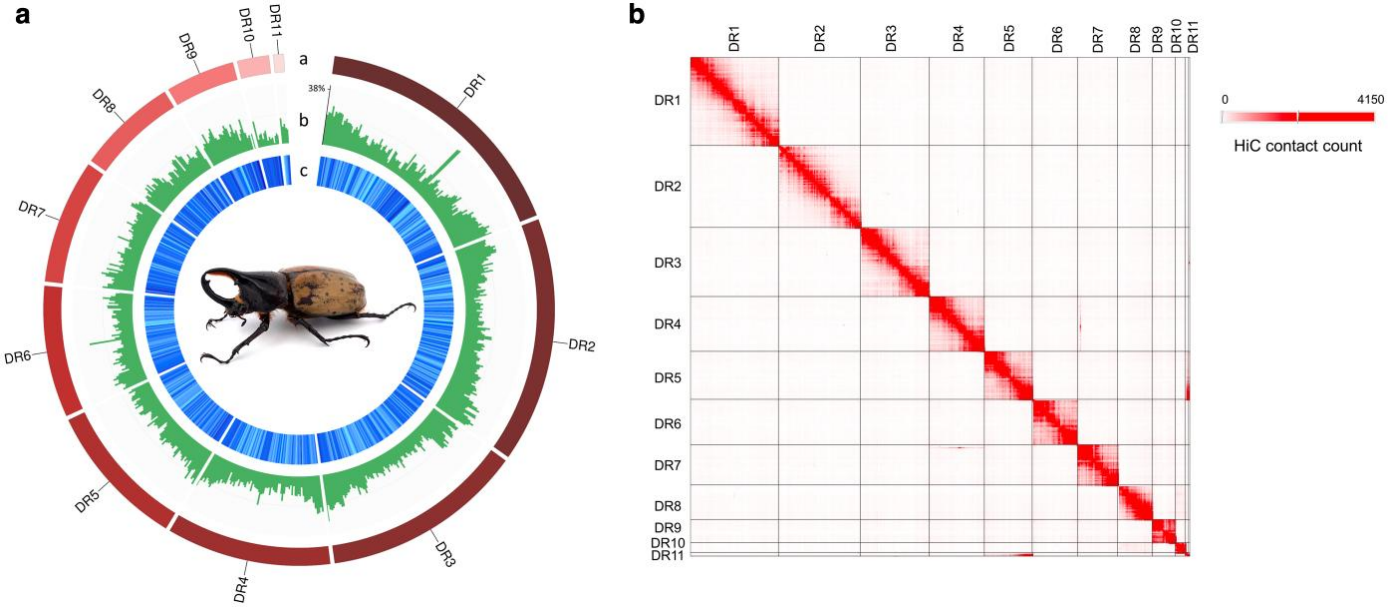
a) Circos plot of the *D. reidi* genome assembly across all 11 pseudochromosomes (DR1−DR11). From the outermost ring to the innermost: a) Pseudochromosome identifiers (from DR1 to DR11), each segment representing a single scaffold or linkage group. b) GC content in percentage plotted as green histogram bars. c) Gene density represented in shades of blue, where lighter cyan indicates lower gene density and darker blue indicates higher density across genomic windows. An adult male of *D. reidi*, which was the sample used for the reference genome sequencing and assembling, is shown at the center (image taken by J.-P.H); b) Genome-wide Hi-C link heatmap of the anchored 11 longest pseudochromosomes of *D. reidi.* The plot displays the observed number of Hi-C interactions within and between scaffolds using YAHS (Zhou et al. 2023). Each pixel refers to a 5-kb bin, and the color value indicates the number of valid Hi-C read pairs that contact within and between scaffolds.

One single adult male *D. reidi* (Fig. 1a), captive bred was obtained from the Taiwan Insect Museum (https://taiwan.insect.tw/home.jsp) and selected as a representative individual for generating the high-quality reference genome of the Giant Hercules beetle group. This *D. reidi* progeny originated from the island of Saint Lucia and has been inbred in captivity for over 7 generations in Taiwan. Long-term inbreeding can significantly reduce heterozygosity in this population, which makes it an ideal candidate for genome sequencing and reference genome assembly. Lower heterozygosity simplifies the assembly process by reducing the complexity introduced by allelic variation, allowing for more accurate reconstruction of genomic regions and improved contiguity in the final assembly.

We extracted high molecular weight genomic DNA (gDNA) from leg muscle samples of the *D. reidi* beetle using the Genomic-tip 20/G kit and Qiagen Buffer Set (Qiagen, Germany), following the manufacturer's instructions. For long-read sequencing, 3 DNA libraries were prepared using the KAPA HyperPrep Kit and Oxford Nanopore Technologies (ONT) Ligation Sequencing Kit V14 and sequenced on a MinION devices using ONT R10.4.1 flow cells and the ONT MinKNOW software. In addition, for short-read sequencing, 1 DNA library was prepared and sequenced by Genomics BioSci & Tech (Taipei, Taiwan) using the Illumina NovaSeq 6000 platform. Prothoracic muscle samples from the same beetle individual were used for high-resolution chromosome conformation capture (Hi-C) sequencing using NextSeq 2000 sequencer at the NGS High Throughput Genomics Core (Academia Sinica). The genomic and Hi-C short-read sequencing data were trimmed using the *fastp* v.0.21.0 before further analyses (Chen et al. 2018). Furthermore, 7 tissue types—brain, antenna, head muscle, thoracic muscle, prothoracic muscle, testis, and phallus—were preserved in RNAlater, and RNA was subsequently extracted separately from each using TRIzol Reagent and the PureLink RNA Mini Kit (Invitrogen). Detailed information on the raw sequencing data generated by each platform is provided in Supplementary Table 1.

### Genome sequencing data processing and assembly

We applied complimentary approaches to assemble a reference genome for *D. reidi*. In particular, after performing high-accuracy base-calling on raw ONT sequencing data using Guppy v5.5.3 with configuration file dna_r10.4_e8.1_sup, and filtering out reads shorter than 10 kb with NanoFilt from the NanoPack software suite (De Coster et al. 2018), 952,572 ultralong reads—corresponding to 20.44 Gb (∼25× coverage)—were used to generate a de novo assembly with wtdbg2 (Ruan and Li 2020). Next, the de novo assembly was polished using filtered high-accuracy Illumina short reads (75× coverage) with the POLCA function of the MaSuRCA toolkit (Zimin et al. 2013). Following that, to improve the continuity of the genome assembly, Hi-C sequencing data (41× coverage) was first mapped to the initial genome assembly using bwa-mem (Li, 2013) and then processed sequenced reads into contact pairs using pairtools v.1.1.0 (Open2C et al. 2024), and then the YaHS scaffolding tool (Zhou et al. 2023 ) was used to reconstruct our *D. reidi* genome assembly based on the topological distribution of chromatin interaction signals.

### RNA sequencing and genome annotation

Repeat library of *D. reidi* was acquired using EDTA pipelines v1.8.2 (Ou et al. 2019) and then classified using RepeatModeler (Flynn et al. 2020). After that, all low-complexity regions and unidentified repeats were removed from the library. The whole-genome sequences of the *D. reidi* genome assembly were soft masked using the TE library generated by EDTA and RepeatModeler, with RepeatMasker (Chen 2004) applied for masking. The microsatellites of *D. reidi* genome were identified using the *MISA* tool with default settings (Beier et al. 2017).

We obtained around 79 Gb of raw RNA sequencing data of *D. reidi*, and then QC trimming was carried out using *fastp* v.0.21.0 (Chen et al. 2018). The transcripts were then mapped to the *Dynastes maya* haploid genome assembly using the splice-aware alignment tool STAR (Dobin et al. 2013). The RNA-seq alignments were used as inputs for ab initio gene prediction on the soft-masked genomes using BRAKER2 v2.1.6 (Brůna et al. 2021). We generated a circus plot using shinyCircos (Yu et al. 2018) to visualize the GC content, gene density of the *D. reidi* genome. Following gene prediction by BRAKER2, we identified orthologous gene families between *D. reidi* and *D. maya* using the OrthoFinder package with default parameters (Emms and Kelly 2019).

We applied a local implementation of the Blast2GO software to investigate the function and gene ontology (GO) terms of the predicted genes in the *D. reidi* genome. We used InterProScan v5.29 (Jones et al. 2014) to examine protein domains, motifs, and other protein signatures by searching against several databases, including Pfam, ProDom, PRINTS, SMART, PANTHER, and PROSITE for all genes predicted from BRAKER2. At the same time, the predicted gene set was locally searched against the nonredundant database using Blastp with the e-value set at 10^−5^. The InterProScan and Blastp results were used as input for Blast2GO (Conesa et al. 2005).

### Comparative genomics

The Hercules beetle system can be divided into 2 main species groups, or clades: White and Giant Hercules beetles (Huang 2017). In the previous study, we published high-quality chromosome for 4 White Hercules beetle genomes (Le et al. 2024), in which we generated *D. maya* genome as the reference for other genomes. In this study, we compare the genome structure of 2 genomes represented for the 2 Hercules beetle groups, which are *D. reidi* and *D. maya*. Therefore, we used MCScanX (Wang et al. 2012) to identify collinear blocks of at least 5 genes for those 2 genome assemblies. Genes were compared with the *D. maya* pseudomolecules using Blastp with an e-value cut-off of 1e^−5^, and the number of Blastp hits was set at 20. The collinearity analyses were visualized using SynVisio (Bandi and Gutwin 2020).

### Sex chromosome identification

We applied the findZX pipeline (Sigeman et al. 2022) to identify the sex-linked genomic regions/chromosome in the *D. reidi* genome by using processed whole-genome sequencing (WGS) data from 1 male and 2 female individuals. The male individual is the same one that we used to generate the *D. reidi* genome assembly. The 2 female individuals were siblings of the male used for genome assembly in this study. Briefly, the software first aligned paired-end sequencing data reads from both male and female samples to the genome assembly and then scanned the genome assembly in windows to clarify sex difference signatures in genome coverage. Regions with reduced coverage in males compared to females are indicative of X-linked regions/chromosome. Conversely, regions with lower coverage in females relative to males may present signals the presence of Y-linked regions/chromosome.

## Results and discussion

### A chromosome-level assembly of the *D. reidi* genome

The preliminary genome assembly of *D. reidi* generated from Nanopore long-read sequencing data using wtdbg2 resulted in a total assembly size of 836,829,348 bp, which was assembled into 6,592 contigs. The contig N50 was 1,844,899 bp, and the L50 was 103, indicating a moderately fragmented initial assembly. After error correction using Illumina short-read data and subsequent scaffolding with Hi-C data, the final genome assembly had a slightly increased total length of 837,131,894 bp and consisted of 5,589 scaffolds. This improved assembly showed a markedly higher scaffold N50 of approximately 66.69 Mb and an L50 of 5, reflecting substantial improvement in contiguity of the genome. The GC content of the final assembly was 34.98%. Note that the 11 longest scaffolds were anchored to a total of 670,923,602 bp, representing 80.15% of the assembled genome (Fig. 1). Assembly completeness, assessed with BUSCO (Simão et al. 2015) using the endopterygota_odb10 database (2,124 BUSCOs), showed a high recovery rate of 95.9%, indicating a high-quality genome assembly (Table 1).

**Table 1.** Summary of sequencing, genome assembly, and genome annotation for the *D. reidi* genome.

Sequencing	
Filtered WGS-Nanopore data (Gb)	20.44
Filtered WGS-Illumina data (Gb)	52.65
Filtered Hi-C data (Gb)	29.3
Filtered RNA-seq data (Gb)	37.71
**Genome assembly**	
Total length (bp)	837,131,894
Number of scaffolds	5589
N50 (bp)	66,686,056
L50	5
Longest scaffold (bp)	121,607,430
GC content (%)	34.98
Length of anchored longest 11 scaffolds (bp)	670,923,602
Percentage of anchored longest 11 scaffolds (%)	80.15
BUSCO completeness (%)	95.9
**Genome annotation**	
Number of repeats	1,913,447
Repeat ratio (%)	55.49
Number of predicted genes	23,351
BUSCO completeness (%)	96
Number of genes in anchored longest 11 scaffolds	18663
Percentage of genes in anchored longest 11 scaffolds (%)	79.92
Number of shared genes	17,348
Number of singletons	3,401
Number of genes in orthogroups	22,189
Number of species-specific orthogroups	474
Number of genes with blast hits	16,285
Number of InterPro protein families	4,994
Number of genes with Blast2GO mapping	9,565

### Repetitive elements

Repetitive elements in the *D. reidi* genome were annotated using a combined pipeline of EDTA and RepeatModeler that integrate de novo and homology-based repeat identification approaches. We also identified microsatellite or simple sequence repeats (SSRs) in the *D. reidi* genome. We found that the average length of SSRs was 57 bp, the total SSR count was 141.816, and the total SSR base was 8.130.436, which accounts for less than 1% for the *D. reidi* genome (Supplementary Table 2). Prior to masking with RepeatMasker, we excluded unknown and unclassified repeat families identified by RepeatModeler from the custom repeat library to ensure more reliable repeat classification. The overall proportion of repetitive sequences varied across pseudochromosomes, ranging from 60.17% in DR11 to 81.02% in DR2, indicating substantial heterogeneity in repeat distribution.

DNA transposons were the most abundant class of repeats in the newly assembled *D. reidi* genome, which comprise more than 75% of the total repetitive content in the genome. Among the identified transposons, the TcMar-Mariner, TcMar-Tc1, hAT-Charlie, and hAT-hAT19 superfamilies were the most frequently observed, which is a pattern commonly observed in coleopteran genomes (such as Le et al. 2024). In addition to DNA transposons, long interspersed nuclear elements (LINEs) and long terminal repeat (LTR) retrotransposons contributed 13.69% and 1.68%, respectively, to the total length of the *D. reidi* genome (Supplementary Fig. 1).

While total repeat content did not vary significantly along individual pseudochromosomes (Fig. 1a), we observed localized enrichment of LINEs and/or LTRs in the central regions of several pseudochromosomes (Supplementary Fig. 2). This pattern is consistent with the known tendency of these elements to accumulate in heterochromatic regions, such as centromeres and telomeres, where recombination is suppressed and transposable element (TE) activity is more tolerated (Rho et al. 2007; Fujiwara 2014; Galindo-González et al. 2017). Although the functional annotation of LTRs and other retrotransposons remains limited in beetles and insects more broadly, the observed accumulation of LTR and non-LTR elements may serve as informatic cytogenetic markers to detect heterochromatic regions. These patterns offer important opportunities for future studies on the structural organization of the *D. reidi* genome and more broadly on genome regulation, epigenetic silencing, and chromosome evolution.

### Genome annotation

We use RNA-seq data (a total of 79.2 GB) to support gene prediction and functional annotation in the *D. reidi* genome. Using BRAKER2, a total of 26,937 protein-coding genes were predicted. Of these predicted genes, 22,229 genes were assigned putative protein functions based on InterProScan domain and motif analyses. Additionally, 14,603 genes were further functionally annotated using Blast2GO, which incorporates Gene Ontology (GO) terms and sequence similarity to known proteins (Table 1). Assessment of gene set completeness using BUSCO revealed that 94.3% of the expected single-copy orthologs were present and complete, which again indicate a high-quality and comprehensive gene annotation. The gene density across the pseudochromosomes is shown in Fig. 1a, which demonstrates variation in gene-rich and gene-poor regions that may be associated with euchromatic and heterochromatic domains, respectively.

### Structural variations between the genomes of Giant and White Hercules beetles

**Fig. 2.**
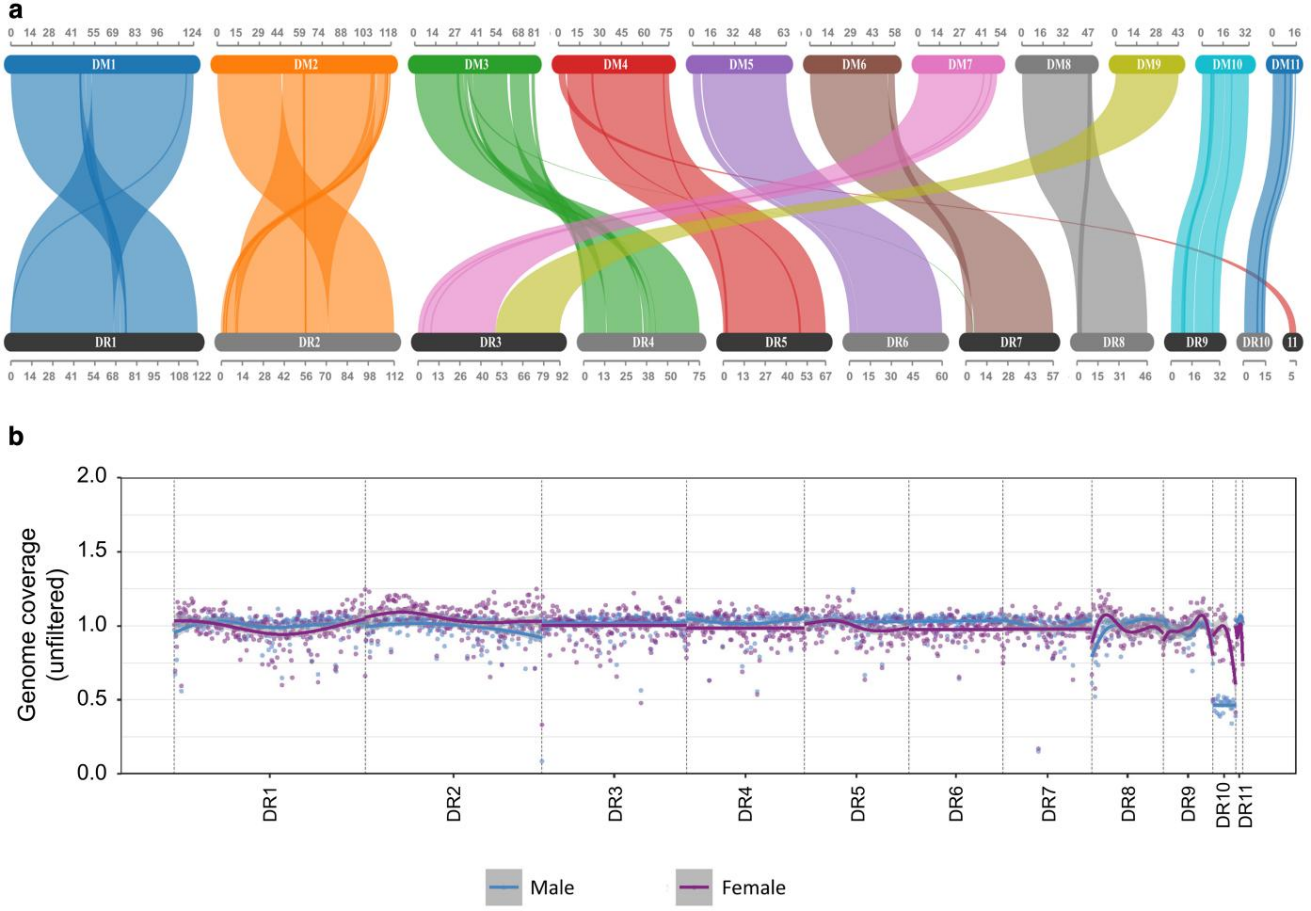
a) Synteny between genetic linkage groups on pseudochromosomes between *D. maya* (upper bars) and *D. reidi* (lower bars). Colored ribbons indicate syntenic blocks shared between species, linking homologous regions across pseudochromosomes (DM1–DM11 and DR1–DR11). Each color corresponds to a pseudochromosome in *D. maya*, with links showing conserved genomic structure and potential chromosomal rearrangements such as translocations or inversions. The scale above and below each bar represents physical distance in megabases (Mb). b) Identification of sex-linked genomic regions in *D. reidi* based on sequencing coverage differences between males and females. Genome coverage (unfiltered) across all 11 pseudochromosomes (DR1 to DR11) in 1-Mb windows. Each point represents mean coverage per window for males (blue) and females (purple), with lines showing overall trends. Note that male samples show approximately 50% reduced sequencing read coverage on pseudochromosome DR10, which is consistent with expectations for an X chromosome in an XY sex-determination system.

To investigate conserved synteny and large-scale structural variation, we conducted a whole-genome comparison between our newly assembled *D. reidi* genome and a previously published genome of *D. maya* (Fig. 2a). Specifically, the genome assembly of *D. maya* has a total length of 729,445,763 bp, with an N50 of 74,954,010 bp, which is a highly contiguous assembly (Le et al. 2024). Furthermore, the 11 longest anchored scaffolds span 721,800,813 bp, accounting for 98.95% of the total assembly, suggesting that most of the genome is represented within these pseudochromosomes (Le et al. 2024).

While both *D. reidi* and *D. maya* belong to the genus *Dynastes* (Huang 2017), they represent distinct lineages: *D. reidi* is part of the Giant Hercules beetle group, whereas *D. maya* belongs to the White Hercules beetle group (Huang 2017). The divergence time between the 2 Hercules beetle groups has been estimated around 3 million years ago (Huang and Knowles 2016). Comparative genome alignment revealed that gene synteny is largely conserved across the 11 pseudochromosomes of both species. However, we also observed apparent chromosomal rearrangements, including several large-scale inversions. For example, a particularly interesting observation from the synteny analysis is that DR5 and DR11 of *D. reidi* show extensive collinearity with different regions of a single pseudochromosome, DM4, in *D. maya*. This pattern strongly suggests a chromosomal fusion event, in which 2 separate ancestral chromosomes (represented in *D. reidi;* Dutrillaux and Dutrillaux 2013) were joined into 1 in the lineage leading to *D. maya.* In contrast, the DR3 in *D. reidi* is fused from DM7 and DM9 in *D. maya*. To check whether misassembly occurred in the scaffolding step, we examined the Hi-C contact map in detail and found that low numbers of Hi-C contacts were detected between DR5 and DR11 compared to the numbers of contacts within the same chromosome, suggesting they are likely separate chromosomes (Supplementary Fig. 3). Additionally, high numbers of Hi-C signals spanned across DR3, supporting that it forms nicely in 1 chromosome (Supplementary Fig. 4). However, we cannot entirely exclude the possibility of misassembly, especially for DR11, which is relatively short and contains many repetitive sequences. Further cytogenetic studies in *D. reidi* are required to confirm our findings.

Furthermore, our results from orthologous gene analysis using OrthoFinder (Emms and Kelly 2019) identified 8,877 single-copy orthologs shared between *D. reidi* and *D. maya*, which possibly reflects the close evolutionary relationship between these 2 species (Supplementary Table 2). Additionally, genomic regions containing genes and gene families previously implicated in male horn morphology and body coloration (Le et al. 2024) were found in syntenic positions between the 2 species (Supplementary Table 3), which indicate the conservation of genomic architecture underlying these phenotypic traits.

### Identifying sex-linked genomic regions

We identified sex-linked genomic regions in the *D. reidi* genome assembly using findZX (Sigeman et al. 2022), a tool designed to detect sex chromosomes by comparing patterns of heterozygosity and read coverage between male and female WGS data. Our results from this analysis revealed marked differences in read depth between sexes on pseudochromosome 10 (DR10), where the male individual exhibited approximately 50% lower read coverage compared to females (Fig. 2b). This consistent pattern implies that DR10 may be the X chromosome or, more conservatively, DR10 may harbor a large proportion of genomic regions linked to the X chromosome. On the other hand, we observed increased coverage in the male sequencing reads relative to females on DR11 (Supplementary Fig. 5), implying potential male-specific sequences; a characteristic feature of Y-linked genomic regions. It is worth noting that the identifying sex chromosomes provide valuable insights into the genetic basis of sex determination and reveal how they evolve across different species and lineages. By applying ultralong-read sequencing technique followed by genome coverage analysis across male and female individuals, we successfully identified the candidate sex-related chromosomes in the *D. reidi* genome. However, in most XY chromosome systems, females typically exhibit no or extremely low read coverage on the Y chromosome. In our case, the read coverage on the candidate Y chromosome was only slightly lower in females than in the male individual. In other words, our observation did not fully match the ideal pattern of near-zero female read coverage on the Y chromosome. This support the idea that sex chromosome evolution is a complex and dynamic process in Hercules beetles (Dutrillaux and Dutrillaux 2013).

When considered alongside the previously described synteny analysis between *D. reidi* and *D. maya*, DR11 emerges as a strong candidate for the Y chromosome in *D. reidi.* Interestingly, our result contrasts with earlier cytogenetic evidence from related species. Specifically, karyotype studies have suggested a neo-Y system in both *D. hercules* (the sister species of *D. reidi*) and *Dynastes tityus* (the sister species of *D. maya*), where sex chromosomes are thought to have originated from fusions between ancestral sex chromosomes and autosomes (Dutrillaux and Dutrillaux 2013). However, our results suggest that in *D. reidi*, the Y chromosome may remain a distinct, unfused chromosome, which is different from the fused autosomal–sex chromosome system observed in *D. maya*. This observation provides new opportunities into studying sex chromosome evolution within Hercules beetles and supports the hypothesis that *D. reidi* may retain a more canonical XY sex determination system, akin to what has been proposed for *Dynastes neptunus* and *Dynastes santa*.

Identifying the Y chromosome in insects is extremely challenging due to its small size, highly repetitive content, degeneration, and lack of gene homology with autosomes (Kaiser and Bachtrog 2010; Huang et al. 2022). These Y-specific genomic features often make both assembly and annotation complicated and difficult. Therefore, further validation is needed to confirm the identity of the candidate Y chromosome in *D. reidi*. Future directions should include cytogenetic karyotyping of *D. reidi*, as well as comparative pangenome analysis of other *Dynastes* species, such as *D. neptunus* and *Dynastes occidentalis* whose karyotype information has been well studied and confirmed previously (Dutrillaux and Dutrillaux 2013), to trace the structural evolution of sex chromosomes across the genus. In addition, improved annotation and characterization of Y-linked genes, both within Scarabaeidae and specifically among Hercules beetles, will be essential for refining our understanding of sex chromosome evolution and their impacts on generating diversity (Bijl and Mank 2025).

## Supplementary Material

jkaf198_Supplementary_Data

## Data Availability

This project has been deposited at NCBI under BioProject: PRJNA815811, BioSample: SAMN46050842, and genome accession: JBNAZT000000000. The annotation and predicted proteins have been deposited in the Dryad data repository (DOI: 10.5061/dryad.fn2z34v7f). Supplemental material available at *G3* online.
