# Supplementary material for: Chromosome-level genome assembly of Dynastes reidi reveals structural evolution of autosomes and the sex chromosomes in Hercules beetles
Source: G3 (Bethesda). 2025 Aug 22;15(11):jkaf198. doi: 10.1093/g3journal/jkaf198 (PMC12611238; doi:10.1093/g3journal/jkaf198)
Supplement: jkaf198_Supplementary_Data [file jkaf198_supplementary_data.pdf]

Supplementary Table 1. Sample collection, voucher, and data availability information for the studied samples

Sample ID	Collection source	Nanopore reads (SRA number)	Illumina reads (SRA number)	Genome assembly (Accession #)
BL_0725	Captive bred adult male	SAMN48387141	HiC sequencing data SAMN48387142<br>Whole genome sequencing SAMN48387143<br>RNA-Seq SAMN48387144	SAMN46050842
BL_0977	Female adult specimen		SAMN48387145	
BL_0978	Female adult specimen		SAMN48387146	

Supplementary Table 2: Statistics of OrthoFinder analysis between the genome assemblies of *Dynastes reidi* (in this study) and *D. maya* (Le et al. 2024)

Number of species	2
Number of genes	48250
Number of genes in orthogroups	44272
Number of unassigned genes	3978
Percentage of genes in orthogroups	91.8
Percentage of unassigned genes	8.2
Number of orthogroups	14970
Number of species-specific orthogroups	1133
Number of genes in species-specific orthogroups	4708
Percentage of genes in species-specific orthogroups	9.8
Mean orthogroup size	3
Median orthogroup size	2
G50 (assigned genes)	3
G50 (all genes)	3
O50 (assigned genes)	4443
O50 (all genes)	5106
Number of orthogroups with all species present	13837
Number of single-copy orthogroups	8877

Supplementary Table 3: Locations of candidate genes that related to the male horn structure and body coloration in *D. reidi* and *D. maya*

Related characters	Candidate genes	Transcript_DR	chr	start	end	Transcript_DM	chr	start	end
Male horn-shape	Ebony	DR_aa13196	DR_5	54928305	54891357	DM_aa9079	DM_4	17800744	17852502
	Ebony	DR_aa13197	DR_5	54993781	54958318	DM_aa9078	DM_4	17746094	17776466
	Ebony	DR_aa23012	DR_5	54928305	54891357				
	yellow-3	DR_aa1076	DR_1	37847820	37846591	DM_aa2341	DM_1	84760455	84761684
	yellow-3	DR_aa1075	DR_1	37829662	37828421	DM_aa2342	DM_1	84778530	84779771
	yellow	DR_aa21627	DR_11	2205796	2220764	DM_aa8679	DM_4	2592323	2581456
	yellow-b	DR_aa16735	DR_7	38121764	38094254	DM_aa13056	DM_6	19720004	19742007
	yellow-g	DR_aa16690	DR_7	36557799	36558920	DM_aa13098	DM_6	21140939	21139429
	yellow-g	DR_aa16689	DR_7	36557799	36558920	DM_aa13099	DM_6	21140939	21139429
	yellow-g	DR_aa25184	DR_1946	19268	17645				
	yellow-g2 precursor	DR_aa24185	DR_1946	19322	25220				
	yellow-e	DR_aa16688	DR_7	36479625	36413494	DM_aa13102	DM_6	21308019	21375935
	yellow-e	DR_aa16688	DR_7	36479625	36413494	DM_aa13103	DM_6	21334891	21375935
	yellow-e3 precursor	DR_aa16687	DR_7	36384093	36409221	DM_aa13104	DM_6	21382219	21379731
	yellow-h	DR_aa16686	DR_7	36338545	36330307	DM_aa13105	DM_6	21439089	21446514
	yellow-x2	DR_aa22414	DR_109	70543	68831	DM_aa13131	DM_6	22337194	22338906
	yellow-x2	DR_aa16662	DR_7	35500774	35499062	DM_aa13131	DM_6	22337194	22338906
	Laccase-2	DR_aa12922	DR_5	43394923	43393597	DM_aa9358	DM_4	29806147	29807470

	Laccase-2	DR_aa12923	DR_5	43592286	43400696	DM_aa9357	DM_4	29611260	29798231
Body coloration	Optix	DR_aa19117	DR_8	45125299	45037815	DM_aa15854	DM_8	676006	676722
	Rax	DR_aa8760	DR_3	74970324	74989458	DM_aa18145	DM_9	18493041	18456363
	Scr	DR_aa14725	DR_6	40806928	40961535	DM_aa11838	DM_5	42976781	43133696
	Scr	DR_aa14726	DR_6	40806928	40961535	DM_aa11839	DM_5	42976781	43133696
	SOX14	DR_aa4298	DR_2	22215013	22218898	DM_aa5670	DM_2	92812779	92802974
	Sp8	DR_aa12958	DR_5	45731919	45639610	DM_aa9316	DM_4	27445525	27540513
	TBX20	DR_aa6958	DR_3	6115395	6203381	DM_aa15583	DM_7	46921086	46912514
	TBX20	DR_aa6960	DR_3		6305636	DM_aa15581	DM_7	46837655	46780004
	TBX20	DR_aa6962	DR_3	6714055	6429364	DM_aa15580	DM_7	46346302	46647480
	TBX20	DR_aa23017	DR_338	32736	40200				
	Eyegone	DR_aa11031	DR_4	62168017	62272773	DM_aa6888	DM_3	13873058	13762169
	PNR	DR_aa7478	DR_3	21210104	21224773	DM_aa15097	DM_7	31361608	31360655
	PNR					DM_aa15096	DM_7	31359815	31347132

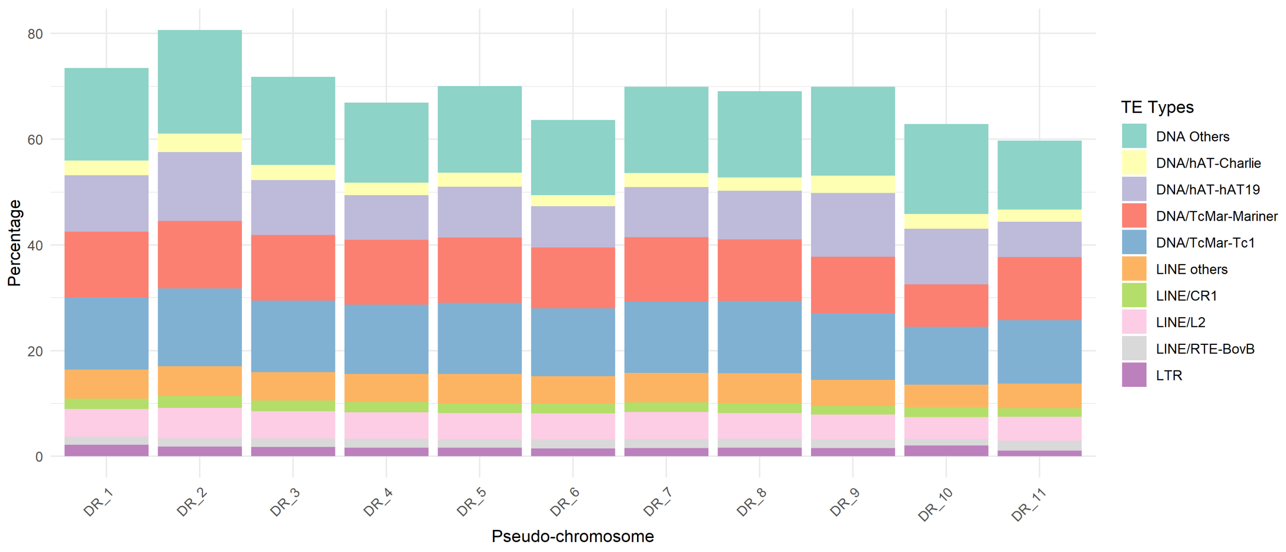

Supplementary Figure 1. Percentage of repeat elements in 11 pseudo-chromosomes of *D. reidi* genome.

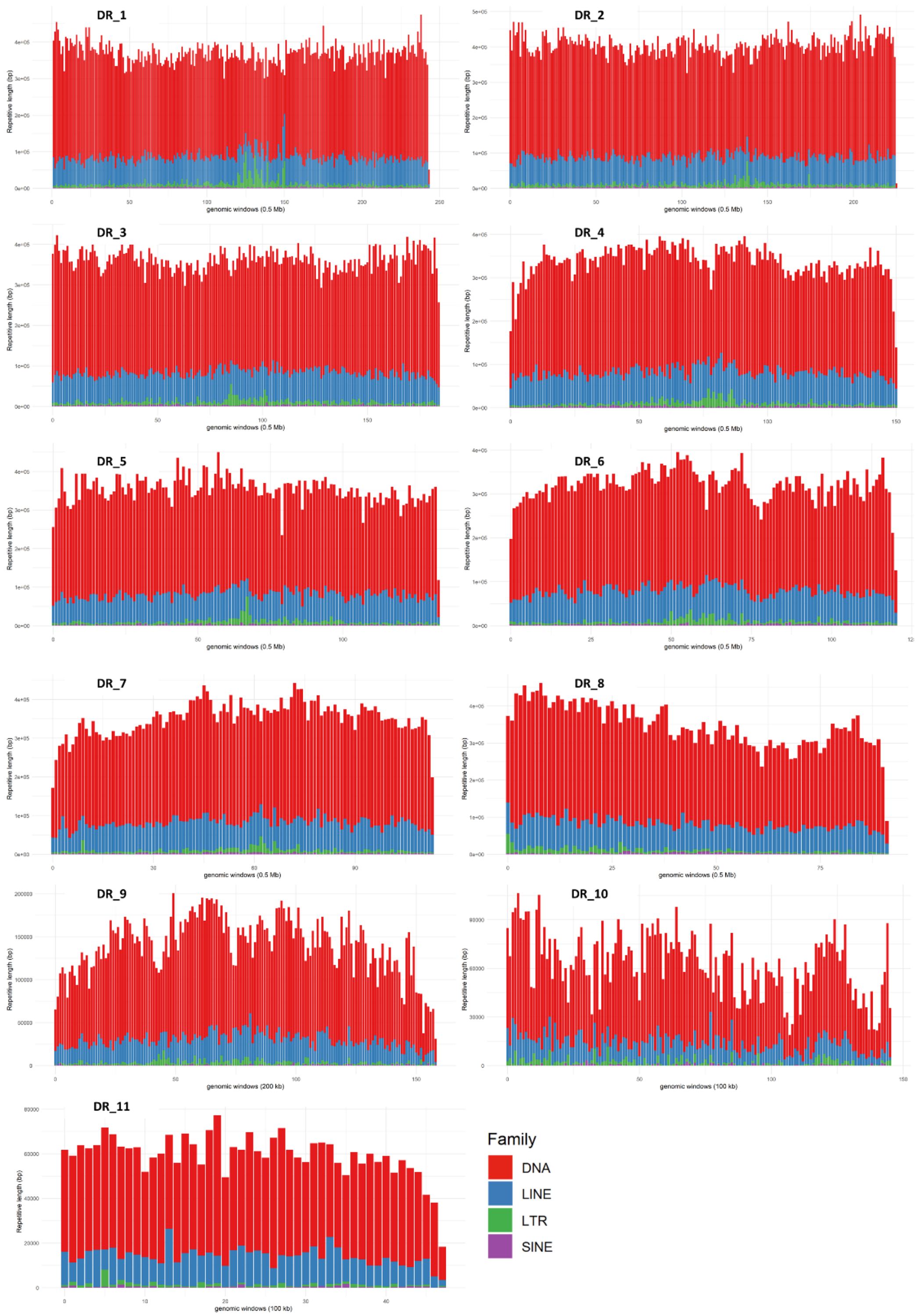

Supplementary Figure 2. Total length of four main repeat element families in 11 pseudo-chromosomes.

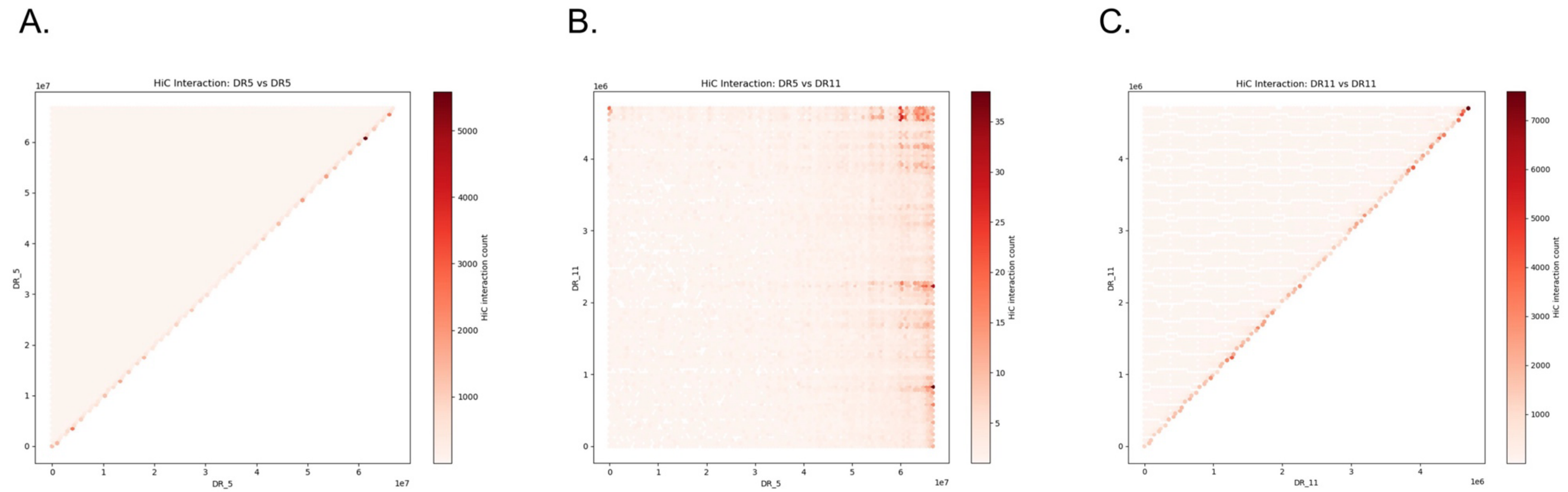

Supplementary Figure 3. Hi-C contact counts at 50kb resolution. A. across DR5 pseudochromosome; B. between DR5 and DR 11; C. across DR11 pseudochromosome.

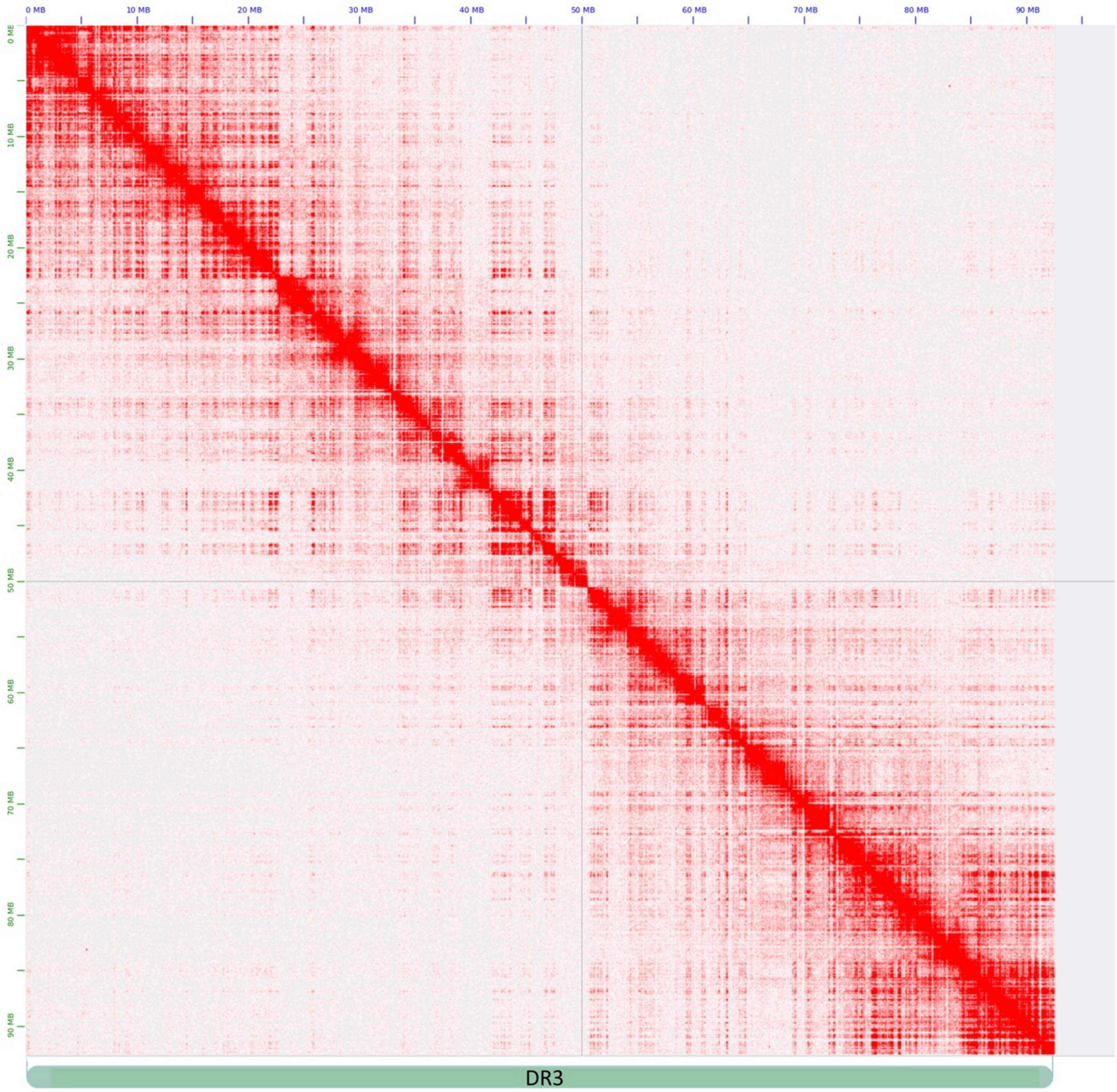

Supplementary Figure 4. Hi-C contact counts spanning across DR3 pseudo-chromosome at 100kb resolution.

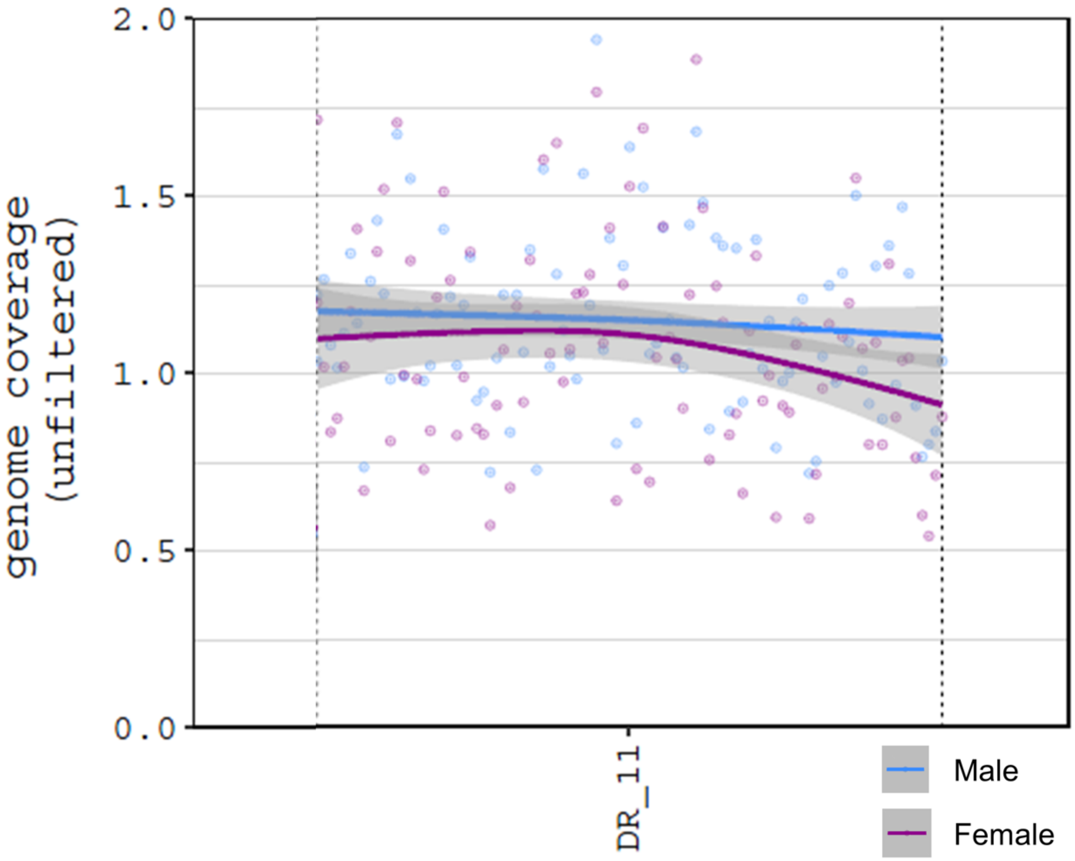

Supplementary Figure 5. Identification of sex-linked genomic regions in *Dynastes reidi* based on sequencing coverage differences between males and females. A zoomed-in view of pseudo-chromosome DR_11 using 50 kb windows, which demonstrates a region with pronounced male-biased increase in coverage. Shaded areas around trend lines indicate 95% confidence intervals.
